# Role of dual specificity phosphatases (DUSPs) in melanoma cellular plasticity and drug resistance

**DOI:** 10.1038/s41598-022-18578-x

**Published:** 2022-08-23

**Authors:** Mithalesh K. Singh, Sarah Altameemi, Marcos Lares, Michael A. Newton, Vijayasaradhi Setaluri

**Affiliations:** 1 Department of Dermatology, School of Medicine and Public Health, University of Wisconsin-Madison, William S. Middleton Memorial Veterans Hospital, Madison, WI 53705 USA; 2 William S. Middleton Memorial Veterans Hospital, Madison, WI 53705 USA; 3 Department of Statistics, Department of Biostatistics and Medical Informatics, University of Wisconsin-Madison, Madison, WI 53706 USA; 4 Department of Dermatology, Wisconsin Institute for Medical Research, University of Wisconsin-Madison, 1111 Highland Avenue, Madison, WI 53706 USA

**Keywords:** Melanoma, Cancer

## Abstract

Melanoma cells exhibit phenotypic plasticity that allows transition from a proliferative and differentiated phenotype to a more invasive and undifferentiated or transdifferentiated phenotype often associated with drug resistance. The mechanisms that control melanoma phenotype plasticity and its role in drug resistance are not fully understood. We previously demonstrated that emergence of MAPK inhibitor (MAPKi)-resistance phenotype is associated with decreased expression of stem cell proliferation genes and increased expression of MAPK inactivation genes, including dual specificity phosphatases (DUSPs). Several members of the DUSP family genes, specifically DUSP1, -3, -8 and -9, are expressed in primary and metastatic melanoma cell lines and pre-and post BRAFi treated melanoma cells. Here, we show that knockdown of DUSP1 or DUSP8 or treatment with BCI, a pharmacological inhibitor of DUSP1/6 decrease the survival of MAPKi-resistant cells and sensitizes them to BRAFi and MEKi. Pharmacological inhibition of DUSP1/6 upregulated nestin, a neural crest stem cell marker, in both MAPKi-sensitive cells and cells with acquired MAPKi-resistance. In contrast, treatment with BCI resulted in upregulation of MAP2, a neuronal differentiation marker, only in MAPKi-sensitive cells but caused downregulation of both MAP2 and GFAP, a glial marker, in all MAPKi-resistant cell lines. These data suggest that DUSP proteins are involved in the regulation of cellular plasticity cells and melanoma drug resistance and are potential targets for treatment of MAPKi-resistant melanoma.

## Introduction

Cutaneous malignant melanoma is a lethal skin cancer. Treatments that target the components of mitogen activated protein kinase signaling (MAPK) pathway, specifically mutated BRAF (mBRAF) and its downstream effector MEK, are highly effective in the short term but only a subset of patients exhibit durable response. Although treatment with a combination of mBRAF and MEK inhibitors (hereafter MAPKi) eliminates most melanoma cells, tumor cells that are either intrinsically resistant or have acquired resistance to MAPKi eventually grow, become aggressive tumors and pose a major clinical challenge. Genetic mechanisms involved in acquisition of MAPKi resistance have been extensively investigated^1^.

There is increasing evidence that phenotypic plasticity of cancer cells and their differentiation along diverse cellular states also contributes to the acquisition of resistance to targeted therapy in various cancers^2^. Cellular plasticity and disrupted differentiation including transdifferentiation are now considered hallmarks of cancer^3^. Molecular mechanisms that regulate phenotypic plasticity and promote survival and proliferation of drug resistant cells are not fully understood^4^.

Lineage plasticity of cutaneous melanoma has been well documented^5^. Melanoma initiating cells (also known as cancer stem cells) form spheroids in vitro and can differentiate into not only melanocytes but also diverse cell types such as adipocytes, chondrocytes and osteocytes^6^. Forced expression of stem cell reprogramming factors in melanoma cells was shown to result in mesenchymal-epithelial transition-like transdifferentiation and attenuated malignancy in vivo^7^. Vasculogenic mimicry of melanoma and its potential as therapeutic vulnerability has been extensively investigated^8^. We showed that a subset of cutaneous primary melanomas exhibits neuronal transdifferentiation and express high molecular weight (mature) microtubule associated protein 2 (MAP2), a marker of neuronal terminal differentiation^9^, and that MAP2 is a potential prognostic marker^10–12^. Recently, using a strategy of reprogramming human melanoma cells to induced-pluripotent cell (iPSC)-like state, we showed that melanoma-derived iPSC-like cells show a tendency to differentiate along mixed- melanocytic and neural- cell lineages, both in vitro and in vivo. These differentiated/transdifferentiated cells also exhibit MAPKi resistance ^13^ suggesting a relationship between signaling pathways that control transdifferentiation and MAPKi-resistance. This notion is supported by the observation that transdifferentiation to neuroendocrine phenotype correlates with treatment resistance in prostate and lung cancers^5,14–16^ and opens the possibility that targeting such pathways can also block or reverse the emergence of new cell lineages and melanoma drug resistance.

Reversible phosphorylation of proteins, regulated by kinases and phosphatases, respectively, is critical to many cellular processes^17^. Protein phosphatases can be classified into three major groups-pSer/Thr-specific phosphatases, pTyr-specific phosphatases and dual-specificity (pSer/Thr and pTyr) phosphatases (DUSPs). Mitogen-activated protein kinase phosphatases (MKPs) are a major subclass of DUSPs. To date, forty-four human DUSPs have been identified^5^. There is accumulating evidence that dysregulation of DUSPs is a common feature of cancers^18^ and DUSPs can serve as prognostic biomarkers^19^. Query of melanoma (SKCM) samples in the Cancer Genome Atlas (TCGA) revealed high mRNA expression of DUSPs in 16.6% tumors. Interestingly, DUSPs are negative regulators of MAPK signaling and are also known to regulate pluripotency and cancer stemness^20,21^. In the context of neural differentiation, DUSP1, an inducible nuclear phosphatase, is of interest. In mice Dusp1 has been shown to play a neuroprotective role in a variety of neurodegenerative disease and *Dusp1*^*-/-*^ mice exhibit defects in neurogenesis^17,22–24^.

Here, we investigated the relationship between DUSPs, neural lineage marker expression and MAPKi-resistance in metastatic melanoma cell lines. Our data show that (a) MAPKi-resistance is associated with loss of expression of neural crest stem cell marker nestin and downregulation neuronal precursor marker MAP2, (b) in MAPKi-resistant cells, inhibition of DUSP1/6 promotes nestin expression and downregulates MAP2, (c) knockdown of DUSP1 or DUSP8 is sufficient to overcome both intrinsic and acquired MAPKi-resistance, and (d) pharmacological inhibition of DUSP1/6 resensitizes resistant cells to MAPKi. Our data suggest that DUSPs provide a link between MAPKi resistance and melanoma transdifferentiation and that targeting DUSPs alone or in combination with MAPKi is a useful strategy to treat MAPKi-resistant metastatic melanoma.

## Material and methods

### Chemicals

BRAF inhibitor, Vemurafenib (PLX4032 #S1267) and MEK inhibitor, Selumetinib (AZD6244 #S1008) were from Selleckchem (Houston, TX). DUSP1 inhibitor BCI [(E)-2-benzylidene-3-(cyclohexylamino)-2,3-dihydro-1H-inden-1-one] (#NSC 150117) was obtained from Axon Medchem (Reston, VA).

### Cell culture

WM series primary and metastatic cells were from Rockland Immunochemicals (Limerick, PA) and cultured in tumor specialized medium (TSM) containing 2% heat inactivated fetal bovine serum (FBS) and 1% penicillin and streptomycin. Matched primary and lymph node metastatic (LNM) cell lines were verified by Short Tandem Repeat (STR) analysis by the Cell Line Authentication Service of UW TRIP Lab. MRA series of metastatic melanoma cell lines (generously provided by Dr. Mark Albertini, Department of Medicine, UW School of Medicine & Public Health, Madison, WI) were genotyped for BRAF and RAS mutations. BRAFi- and MEKi-resistant MRA6 cell lines MRA6BR and MRA6MR, respectively, were generated in vitro by culturing parental MRA6 cells in progressively increasing concentrations of PLX4032 or AZD6244 (1–10 µM) for 3 days at each concentration and allowing surviving cells to proliferate. The resulting MRA6BR and MRA6MR cell lines were maintained medium containing 10 µM BRAFi and MEKi, respectively. Mouse melanoma cells YUMM1.7, YUMM3.3, and YUMMER 1.7 were generously provided by Dr. Marcus Bosenberg, Yale University School of Medicine, New Haven, CT). All MRA series and mouse melanoma cell lines were cultured in DMEM, 10% fetal bovine serum (FBS), and 1% penicillin and streptomycin. All cells were cultured in a humid incubator at 37 °C with 5% CO_2_ and were regularly tested for mycoplasma. Details of all the cell lines used in this study are listed in Supp Table S1.

### Antibodies

Anti-DUSP1, -DUSP3, -DUSP8, and -DUSP9 antibodies were obtained from Abcam (Boston, MA). Antibodies for Nestin, MAP2, GFAP, p38 MAPK, phospho-p38 MAPK, ERK1/2, pERK1/2, JNK1, and phospho-SAPK/JNK (Thr183/Tyr185) were obtained from Cell Signaling (Danvers, MA). Anti-GAPDH antibody was obtained from Proteintech (Rosemont, IL) (See Table S2).

### MTT assays

In a flat-bottomed 96-well plate, 5000 cells/well were plated, incubated at 37 °C overnight and then treated with various chemical agents for the times specified in the legends to the figures. At the endpoint, 20 µl of 5 mg/ml Thiazolyl Blue Tetrazolium Bromide (MTT; Sigma-Aldrich) was added to the wells and plates were incubated for 1 h at 37 °C, and absorbance at 540 nm was measured using a Biotek Synergy H1 Multi-Mode Plate Reader.

### Western blotting

Cells were harvested and lysed in RIPA buffer containing Halt protease inhibitor cocktail (Thermo Fisher Scientific) and phosphatase inhibitor cocktail (Thermo Fisher Scientific). The samples were then sonicated, centrifuged for 30 min at 4 °C and the supernatants were collected. The protein concentration was determined using the Pierce BCA Protein Assay Kit (Thermo Fisher, #23227). SDS-PAGE was performed approximately with 30 µg of protein, and proteins were transferred to a PVDF membrane. The membrane was blocked with 5% nonfat dry milk prepared in TBST buffer. After overnight incubation with primary antibodies at optimized dilutions (Table S2), membranes were washed and incubated with HRP conjugated secondary antibodies. Alternatively, blots were incubated with primary antibodies to proteins of interest together with GAPDH. Protein bands were detected using ECL Start Western Blotting Detection Reagent (Thermo Fisher, #32106) and imaged on an LICOR Fc Odyssey. (LI-COR Biotechnology Lincoln, Nebraska) and the band intensities were quantified using ImageJ (NIH).

### DUSP1 and DUSP8 siRNA knockdown

Equal number of cells (2.5 × 10^5^ cells/mL) were plated in a 6-well plate to a confluence of 60–90% 24 h before transfection. Lipofectamine™ RNAiMAX (#13778075, Thermo Fisher Scientific) was used to transfect cells with a green fluorescent protein control siRNA (#EHUEGFP) or DUSP1 (#EHU016821) or DUSP8 (#EHU118201) esiRNAs (Sigma, USA). The transfection mixture was prepared in antibiotic- and reduced serum media (Opti-MEM) and after 6 h of transfection, medium was changed, and the cells were harvested after 72 h.

### RNA-Seq analysis

FastQC (Andrews 2010, http://www.bioinformatics.babraham.ac.uk/projects/fastqc/) was used for quality control; no samples were omitted from the analysis. Using Bowtie 0.12.8^26^, sequence reads were mapped against the Human genome (Hg19 Refseq reference), with up to two mismatches and up to 20 multiple hits allowed. The expected transcripts per million (TPMs) were calculated using RSEM 1.2.3^27^. EBSeq^31^ were used to identify DUSPs genes between datasets of MPKi-resistant (MRA5, MRA6BR, MRA6MR) and MAPKi-sensitive (MRA6) cell lines, matched primary and lymph node metastatic (LNM) cell lines, publicly available datasets GSE24862^25^ and single RNAseq GSE116237^26^. We focused on DUSPs genes with a probability of differential expression of at least 0.99.

### Gene expression profiling interactive analysis (GEPIA2)

The overall survival (OS) and DUSPs mRNA expression in melanoma in TCGA-SKCM datasets was analyzed using GEPIA2 (http://gepia.cancer-pku.cn/index.html), an online tool for visualization, and evaluation of large-scale cancer-related genomics data sets^27^. The survival map was generated by GEPIA2 to screen the prognostic value of DUSPs genes in melanoma, with 0.05 as the significance level and "Median" as the group cutoff.

### TCDA (The cancer druggable gene atlas)

A recent published study by Jiang et al. generated a comprehensive blueprint of potential druggable genes (PDGs) across cancers through a systematic interaction of the druggable genome and the cancer genome^28^. The Cancer Druggable Gene Atlas (TCDA) portal (http://fcgportal.org/TCDA/) was used to query whether DUSPs are predicted to be druggable. Details of all the DUSPs as a potential druggable genes are listed in Supp Table S3.

### Statistical analysis

All statistical analyses were performed using the Analysis function of GraphPad Prism9 (Ver. 9.2.0). Differences with *p* values < 0.05 were considered statistically significant**.**

## Results

### Gene expression profiles of drug resistant melanoma cells

In a previous study aimed at understanding the relationship between melanoma plasticity and MAPKi resistance, we performed whole transcriptome sequence (RNAseq) analysis of MAPKi- sensitive and MAPKi-resistant (both intrinsic and acquired) melanoma cell lines^13^. Among these cell lines, we noted an inverse relationship between expression of genes in the GO term MAPK regulatory pathways and the GO term stem cell proliferation pathways. Upregulation of genes in the MAPK regulatory pathway was associated with drug resistant phenotype. A further analysis of MAPK regulatory genes showed that mRNAs for several DUSPs-DUSP1 DUSP5, DUSP8, DUSP10 and DUSP14-were upregulated in intrinsically MAPKi-resistant melanoma cell line (Supp Fig. S1A).

### Expression of DUSP proteins during melanoma tumor progression

**Figure 1:**
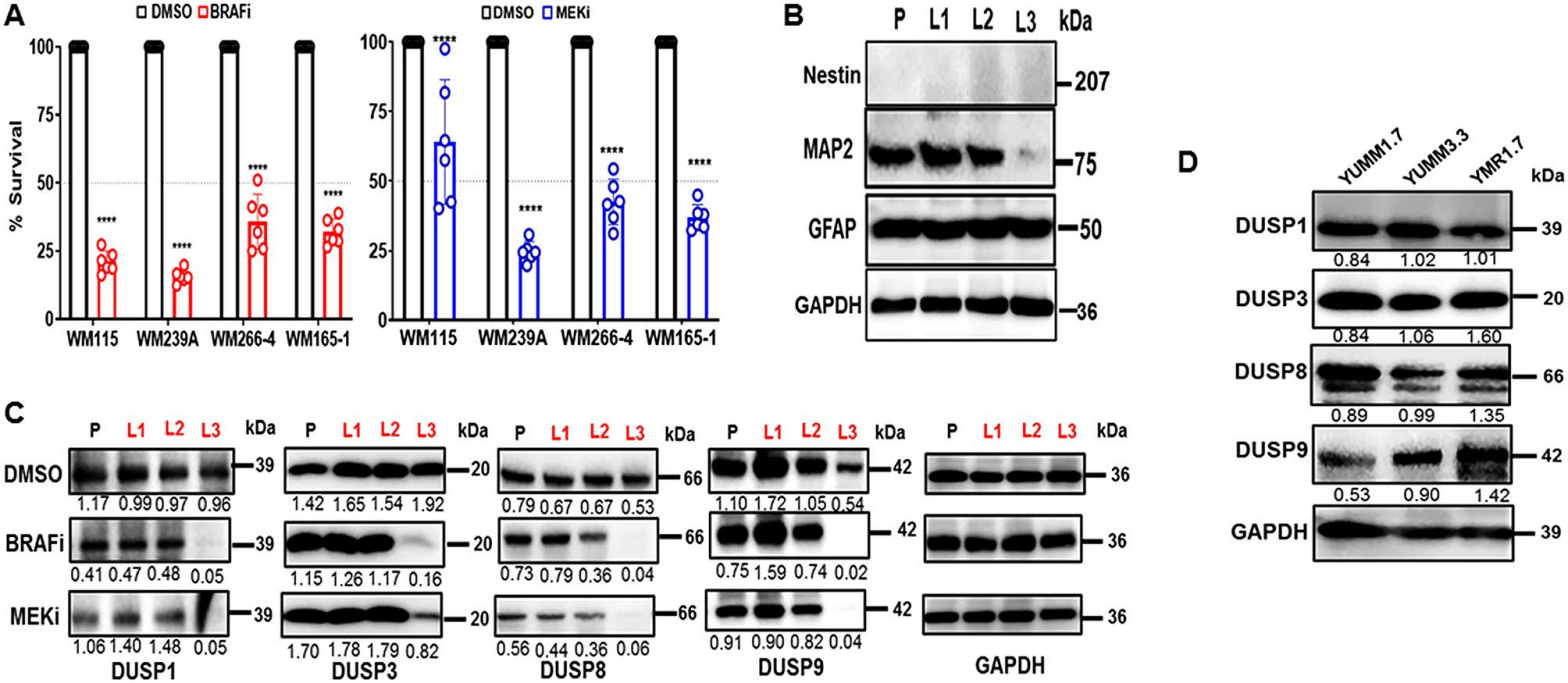
Expression of DUSPs during melanoma progression and the effect of BRAFi and MEKi. (**A**) Effect of BRAFi (PLX4032) or MEKi (AZD6244) on the survival of primary and lymph node metastatic cell lines derived from same patient. Five thousand cells were plated in 6 replicates in 96-well plates and treated with 10 μM PLX4032 or AZD6244 for 72 h and cell survival estimated by MTT assay. Data were analysed by Student t-test and shown as mean ± SD.  ∗Indicates *P* ≤ 0.05;   ∗  ∗  < 0.01;   ∗∗ ∗  < 0.001;  ∗  ∗ ∗∗  < 0.0001. (**B**) Western blot analysis showing endogenous levels of Nestin, MAP2, and GFAP proteins in genetically matched primary (P) WM115 and lymph node metastatic cell lines WM239A (L1), WM266-4 (L2) and WM165-1 (L3). GAPDH levels were used to monitor protein loading. Numbers on the right indicate the molecular (kDa) of the respective proteins. (**C**) Western blot analysis showing endogenous levels of DUSP1, DUSP3, DUSP8 and DUSP9 proteins in genetically matched primary (P) WM115 and lymph node metastatic cell lines WM239A (L1), WM266-4 (L2) and WM165-1 (L3), and after 48 h treatment with DMSO or 10 μM BRAFi (PLX4032) or MEKi (AZD6244). GAPDH levels were used to monitor protein loading. Numbers on the right indicate the molecular (kDa) of the respective proteins. Band intensities were measured using ImageJ and numbers below each band indicate relative expression normalized to GAPDH. (**D**) Expression of DUSP1, DUSP3, DUSP8 and DUSP9 in mouse melanoma cell lines YUMM 1.7 (*Braf*^*V600E/wt*^* Pten*^*−/−*^* Cdkn2*^*−/−*^) and YUMM 3.3 (*Braf*^*V600E/wt*^* Cdkn2*^*−/−*^) YUMMER1.7 is a single cell-derived clone generated from YUMM1.7 irradiated with three rounds of UVB radiation (REF). GAPDH levels were used to monitor protein loading. Numbers on the right indicate the molecular (kDa) of the respective proteins. Band intensities were measured by ImageJ and relative expression normalized GAPDH is shown.

Whole transcriptome analysis of a set of matched primary and lymph node metastatic (LNM) melanoma cell lines established from the same patient showed variable expression of several DUSPs (Supp Fig. S1B). We investigated whether DUSPs are regulated during melanoma tumor progression by evaluating expression of DUSP proteins in this set of matched primary and metastatic melanoma cell lines. WM115 was derived from a VGP primary tumor, and WM266-4, WM239A, and WM165-1 were derived from three lymph node metastases (LNM) that were reported to have arisen progressively distant anatomical locations from the VGP primary melanoma^29^. Short tandem repeat (STR) genotyping confirmed that these four cell lines originated from the same patient. These matched cell lines harbor oncogenic BRAF(V600E) mutation and are sensitive to BRAFi and MEKi (Fig. 1A).

As shown in Fig. 1B, in the matched series of primary and LNM cell lines, MAP2 (microtubule associated protein 2), a neuronal differentiation marker, is expressed abundantly in the primary and early LNM but not in the distant LNM WM165-1 cells whereas GFAP (glial fibrillary acidic protein), a glial cell marker is present in all cell lines. Neural crest stem cell (NCSC) marker nestin, on the other hand, was not detectable in any of these cell lines.

All four DUSPs tested (DUSP1, -3, -8 and -9) are present in both primary and LN metastatic cell lines (Fig. 1C). Interestingly, DUSP1, -8 and -9 appear to be downregulated and DUSP3 is upregulated in LNM WM165-1 compared to primary melanoma and other LNM derived cells. All four DUSPs tested are also found in mouse melanoma cell lines YUMM1.7, YUMM3.3 and YUMMER1.7, a variant YUMM1.7 exposed to UV radiation (Fig. 1D), with Dusp9 showing upregulation in the YUMMER1.7 cell line.

We investigated whether DUSP expression in these matched primary and LNM cell lines is related to MAPK signaling. Treatment with BRAF(V600E) inhibitor (BRAFi) vemurafenib/PLX4032 or MEK inhibitor (MEKi) selumatenib/AZD6244 had no marked effect on expression of the DUSPs, except in LNM cell line WM165-1, where expression of all four DUSPs was significantly downregulated by both BRAFi and MEKi treatment (Fig. 1C). These data suggest that regulation of DUSP by MAPK signaling is a feature of advanced metastatic melanoma.

### Relationship between MAPKi-resistance and melanoma transdifferentiation

To investigate the role of DUSPs in transdifferentiation of metastatic melanoma cells and their response to MAPKi, we selected two BRAF(V600E) mutant human metastatic melanoma cell lines––MRA5 is intrinsically resistant to both BRAFi and MEKi, while MRA6 is sensitive to both inhibitors. Cell lines MRA6BR and MRA6MR with acquired resistance to BRAFi and MEKi, respectively, were derived in vitro from the MAPKi-sensitive MRA6 cells^30^.

**Figure 2:**
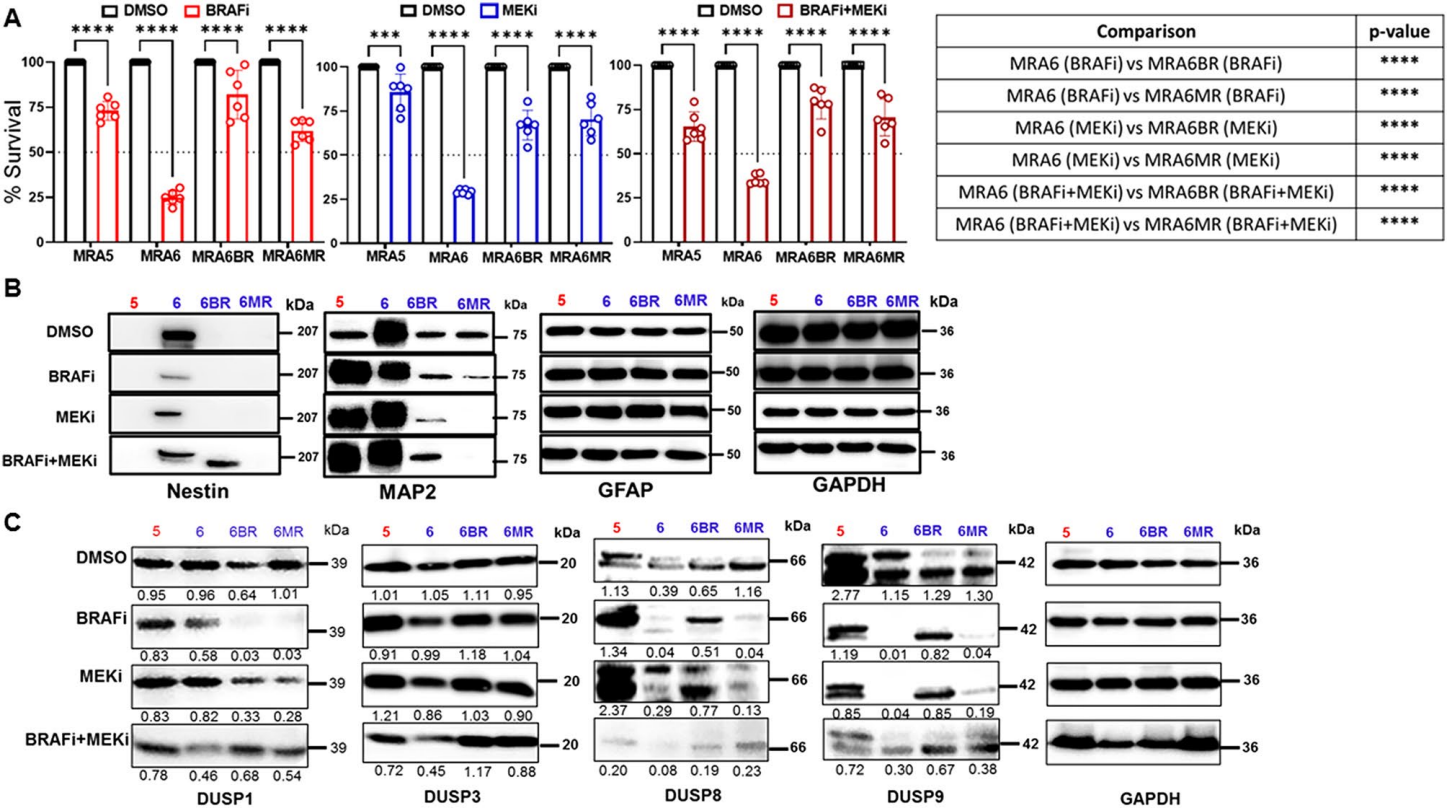
Expression of neural markers and DUSPs in MAPKi-sensitive and -resistant metastatic melanoma cells. (**A**) Effect of BRAFi (PLX4032), MEKi (AZD6244) alone or combination of BRAFi + MEKi on survival of intrinsically MAPKi-resistant MRA5, MAPKi-sensitive MRA6 cell lines and BRAFi-resistant MRA6BR and MEKi-resistant MRA6MR metastatic melanoma cells. Cells were plated in 6 replicates in 96-well plate and treated with DMSO or 10 μM BRAFi PLX4032 or MEKi AZD6244 or combination of 5 µM BRAFi and 5 µM MEKi for 72 h. Data shown as mean ± SD were analyzed by Student’s t-test.  ∗Indicates *P* ≤ 0.05;   ∗ ∗  < 0.01;  ∗ ∗ ∗  < 0.001;  ∗ ∗ ∗∗  < 0.0001. (**B**) Western blot analysis showing the expression of neural stem cell marker Nestin, and neural differentiation markers MAP2 and GFAP proteins after 48 h treatment with DMSO or 10 μM BRAFi or MEKi or combination of BRAFi and MEKi. GAPDH levels were used to monitor protein loading. Numbers on the right indicate the molecular (kDa) of the respective proteins. Band intensities normalized to GAPDH expression using ImageJ are shown. (**C**) Western blot analysis showing expression of DUSP1, DUSP3, DUSP8 and DUSP9 proteins after 48 h treatment with DMSO or 10 μM BRAFi or MEKi or combination of BRAFi and MEKi. GAPDH levels were used to monitor protein loading. Numbers on the right indicate the molecular (kDa) of the respective proteins. Band intensities normalized to GAPDH expression using ImageJ are shown.

As shown in Fig. 2A, treatment with 10 µM BRAFi or MEKi alone, or a combination of BRAFi + MEKi (5 µM each) decreased the survival of MAPKi-sensitive MRA6 cells by approximately 70% whereas the survival of the intrinsically MAPKi-resistant MRA5 and the MRA6BR and MRAMR cells decreased only by 25%.

Interestingly, expression of the neural crest stem cell (NCSC) marker nestin is detectable only in the MAPKi-sensitive MRA6 cells but not in the MAPKi-resistant cells (Fig. 2B) consistent with our data on whole transcriptome RNAseq analyses^13^. Nestin expression in the MAPKi-sensitive MRA6 cells was downregulated in response to treatment with BRAFi or MEKi alone, or combination of BRAFi and MEKi. Treatment with the combination of BRAFi and MEKi upregulated nestin expression only in melanoma cells with acquired resistance to BRAFi but not to MEKi.

MAP2, the low molecular weight (70 kDa) form, a marker of immature neurons, is found in all cell lines with the most abundant expression in the MAPKi-sensitive MRA6 cells showing that MAPKi-sensitive cells exhibit both NCSC marker nestin and the neuronal differentiation marker, MAP2. MAPKi treatment had no effect on MAP2 expression in MRA6 cells but resulted in upregulation of this neuronal marker in intrinsically resistant MRA5 cells and its downregulation in MAPKi-resistant MRA6BR and MRA6MR cells. In contrast to the stem cell and neuronal marker, all cell lines expressed the glial differentiation marker GFAP irrespective of their sensitivity to MAPKi and GFAP expression was not altered by MAPKi treatment. These findings support the notion that neuronal differentiation in melanoma is associated with development of MAPKi resistance.

### Relationship of DUSPs to drug resistance

Next, we analyzed the expression of DUSP1, -3, -8 and-9 proteins in intrinsically MAPKi-resistant MRA5 and MAPKi-sensitive MRA6 cell lines, and MRA6 cells with acquired resistance to BRAFi-(MRA6BR) and to MEKi (MRA6MR) grown in the absence or presence of BRAFi (PLX4032) or MEKi (AZD6244).

As shown in Fig. 2C, DUSP1 was detectable in all four cell lines in the absence of MAPKi. Treatment with 10 µM BRAFi or MEKi alone resulted in a marked downregulation of DUSP1 in MAPKi-resistant MRA6BR and MRA6MR cells but not in the parental MAPKi-sensitive MRA6 cells.

Similar to DUSP1, nearly similar levels of DUSP3 were found in all MAPKi-sensitive (MRA6) and MAPKi-resistant lines (MRA5, MRA6BR, and MRA6MR) lines. Treatment with BRAFi or MEKi alone did not cause marked change in DUSP3 expression, whereas treatment with combination of BRAFi and MEKi appeared to downregulate DUSP3 in both MRA5 and MRA6 cells.

Varied levels of DUSP8 expression was noted with higher expression in MRA5 and MRA6MR cells compared to MRA6 and MRA6BR cells. Treatment with BRAFi or MEKi markedly inhibited DUSP8 expression in MAPKi-sensitive MRA6 cells compared to MAPKi-resistant MRA5 cells. Treatment with BRAFi or MEKi reduced DUSP8 expression in MEKi-resistant MRA6MR cells similar to the parental MAPKi sensitive MRA6 cells.

Most abundant expression of DUSP9 is detectable in the MAPKi-resistant MRA5 cells compared to MAPKi-sensitive MRA6 and its MAPKi-derivatives MRA6BR, and MRA6MR. Interestingly, treatment with BRAFi or MEKi alone or with a combination of both caused marked downregulation of DUSP9 only in MAPKi-sensitive MRA6 and its MEKi-resistant MRA6MR cells compared to the intrinsically MAPKi-resistant MRA5 and the BRAFi-resistant MRA6BR. Overall, the pattern of expression of DUSPs tested in this study and the effect of BRAFi and MEKi on their expression suggest a varied relationship between DUSPs and MAPKi resistance.

### Role of DUSP1 and DUSP8 and MAPKi-resistance

**Figure 3:**
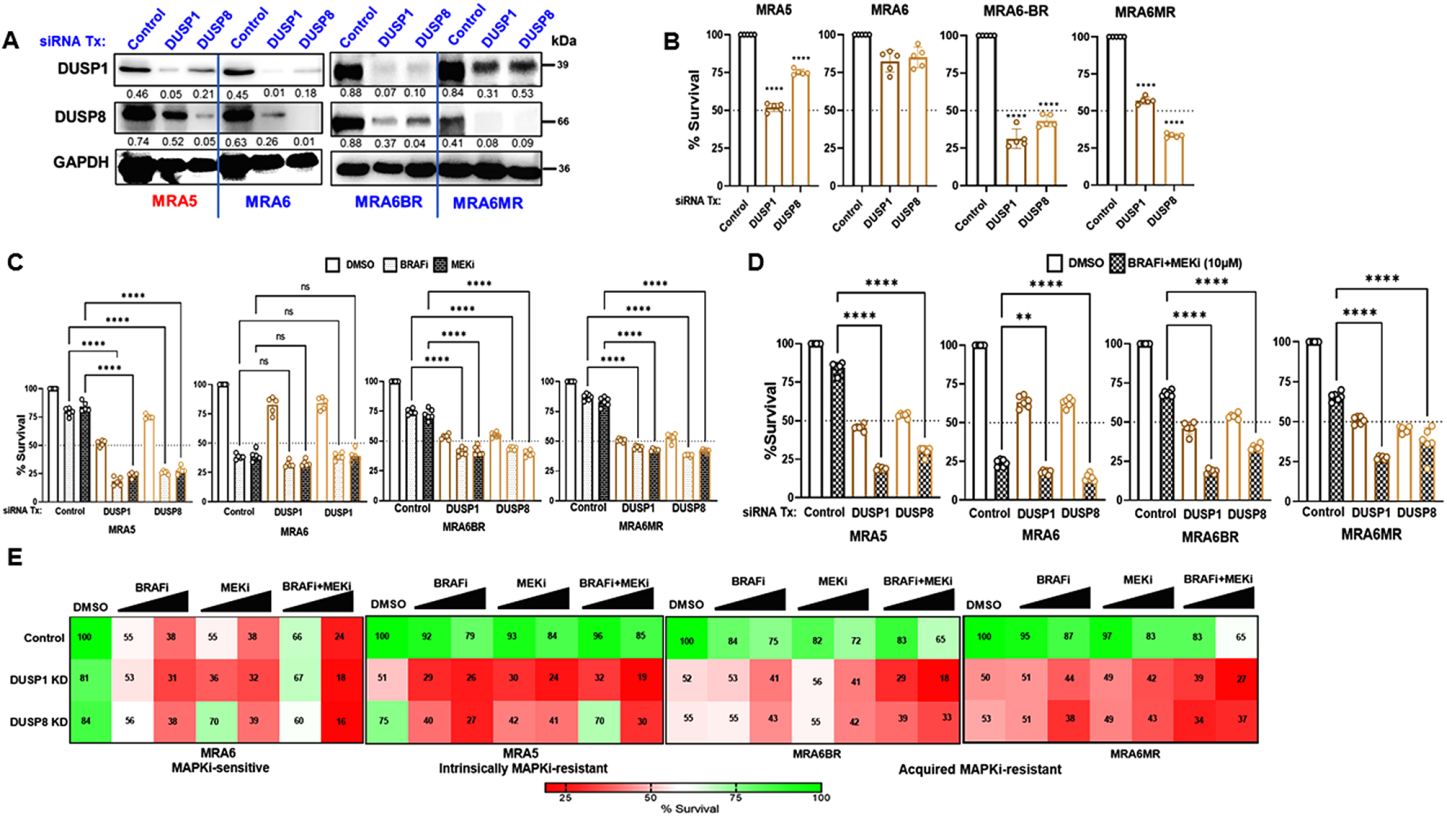
Effect of DUSP1 and DUSP8 knockdown on MAPKi-sensitivity. (**A**) Intrinsically MAPKi-resistant MRA5, MAPKi-sensitive MRA6 cells and MRA6 cells with acquired resistance MRA6BR, MRA6MR cells were transfected with DUSP1 and DUSP8 Misson® esiRNAs or control siRNA, harvested at 72 h and western blot analysis was performed using anti-DUSP1 and DUSP8 antibodies. GAPDH was used as loading control. (**B**) Survival of DUSP1 (dark brown bars) or DUSP8 (light brown bars) siRNA transfected cells was assessed by MTT assay and data are shown as percent survival of control siRNA transfected cells. (**C**) MAPKi-sensitive and MAPKi-resistant cells were transfected with DUSP1 and DUSP8 siRNAs. Cells were trypsinized 24 h post-transfection and 5000 cells were seeded in 96-well plates in 5–6 replicate wells and treated for 48 h with DMSO or 10 µM PLX4032 or 10 µM AZD6244. (**D**) MAPKi-sensitive and MAPKi-resistant melanoma cells were transfected with DUSP1 and DUSP8 siRNAs and treated with DMSO or combination of PLX4032 + AZD6244 as described above in (C). Data are shown as mean ± SD and the data were analyzed using Student’s t-test. *P* values: * denotes *P* ≤ 0.05, ** ≤ 0.01; *** ≤ 0.001 and **** ≤ 0.0001. Differences in survival between DMSO treated control siRNA transfected and DMSO treated DUSP siRNA group were all statistically significant (*P* values: **** ≤ 0.0001). To minimize crowding of the graphs with lines, these comparisons are not shown but shown in Suppl Fig. 2. For comparison, dotted line shows 50% survival. (**E**) MAPKi-resistant and MAPKi-sensitive melanoma cells were transfected with DUSP1 and DUSP8 siRNAs. Cells were trypsinized 24 h post-transfection and 5000 cells were seeded in 5–6 replicate wells in 96-well plates and treated for 48 h with DMSO or 2.5 µM PLX4032, AZD6244 or 10 µM PLX4032, AZD6244 (MRA5 and MRA6 cells). Or combination of PLX4032 + AZD6244. Data are shown as mean ± SD and analyzed using Student's t-test. *P* values: * denotes *P* ≤ 0.05, ** ≤ 0.01; *** ≤ 0.001 and **** ≤ 0.0001.

To understand the role of DUSPs in melanoma cell survival, we used siRNA-mediated knockdown (KD) of DUSP1 or DUSP8, employing MISSION® esiRNAs (Millipore Sigma), which are endoribonuclease prepared heterogeneous pools of siRNA that all target the same mRNA sequence. These multiple silencing triggers are reported to lead to highly specific and effective gene silencing. These siRNAs efficiently reduced the levels of respective targets (Fig. 3A and Supp Fig. S2A). DUSP1 KD reduced the survival of MAPKi-resistant MRA5, MRA6BR and MRA6MR cells by 45, 75, and 40%, respectively, whereas DUSP8 KD reduced their survival by 25, 55, and 70%, respectively. Interestingly, DUSP1 or DUSP8 KD had only modest effect (20% and 15% decrease, respectively) on the survival of MAPKi-sensitive MRA6 cells (Fig. 3B). These data indicate that DUSP1 and DUSP8 contribute to the survival MAPKi resistant cells.

Next, we investigated the effect of DUSP1 and DUSP8 KD on MAPKi sensitivity of melanoma cells. We transfected MAPKi-resistant MRA5 and MAPKi-sensitive MRA6 metastatic melanoma cells with control or DUSP1 or DUSP8 siRNA and then treated the cells with 10 μM BRAFi or MEKi alone or in combination. As shown in Fig. 3C, DUSP1 or DUSP8 KD sensitized the drug resistant MRA5, MRA6BR and MRA6MR cells to MAPKi, i.e., compared to < 20–25% decrease in survival of control siRNA transfected cells, treatment of DUSP1 and DUSP8 KD cells with BRAFi or MEKi caused > 50–75% decrease in cell survival. On the other hand, DUSP1- or DUSP8-KD had no effect on the response of the MAPKi-sensitive MRA6 cells to MAPKi. When treated with a combination BRAFi and MEKi), all MAPKi-resistant cell lines (MRA5, 6BR, 6MR) with DUSP1- and DUSP8-KD showed a significant decrease (> 50–80%) in survival compared to BRAFi + MEKi treated control siRNA transfected cells. Likewise, DUSP1- or DUSP8-KD had a small but significant effect on the survival MAPKi-sensitive MRA6 cells (Fig. 3D). Statistical analyses of data shown in Fig. 3C,D are shown in Supp Fig. S2B,C.

DUSP1 or DUSP8 KD also sensitizes MAPKi-resistant MRA5 and MRA6BR, MRA6MR to MAPKi resulting in a dose-dependent decrease in survival upon treatment with BRAFi or MEKi alone or in combination. MAPKi-sensitive MRA6 cells transfected with control siRNA show a dose-dependent decrease in survival, but neither DUSP1-or DUSP8 KD altered their response to MAPKi treatment (Fig. 3E) although they were treated with identical concentrations of BRAFi, MEKi or combination of BRAFi and MEKi as their resistant counterparts.

These findings suggest that MAPKi-resistant (MRA5, MRA6BR, MRA6MR) melanoma cells are more dependent on DUSP1 and DUSP8 for their survival than MAPKi-sensitive (MRA6) cells. These findings also point to a role for DUSP1 and DUSP8 in melanoma MAPKi-resistance.

### Effect of DUSP1/6 inhibition on the MAPK activity and melanoma transdifferentiation

**Figure 4:**
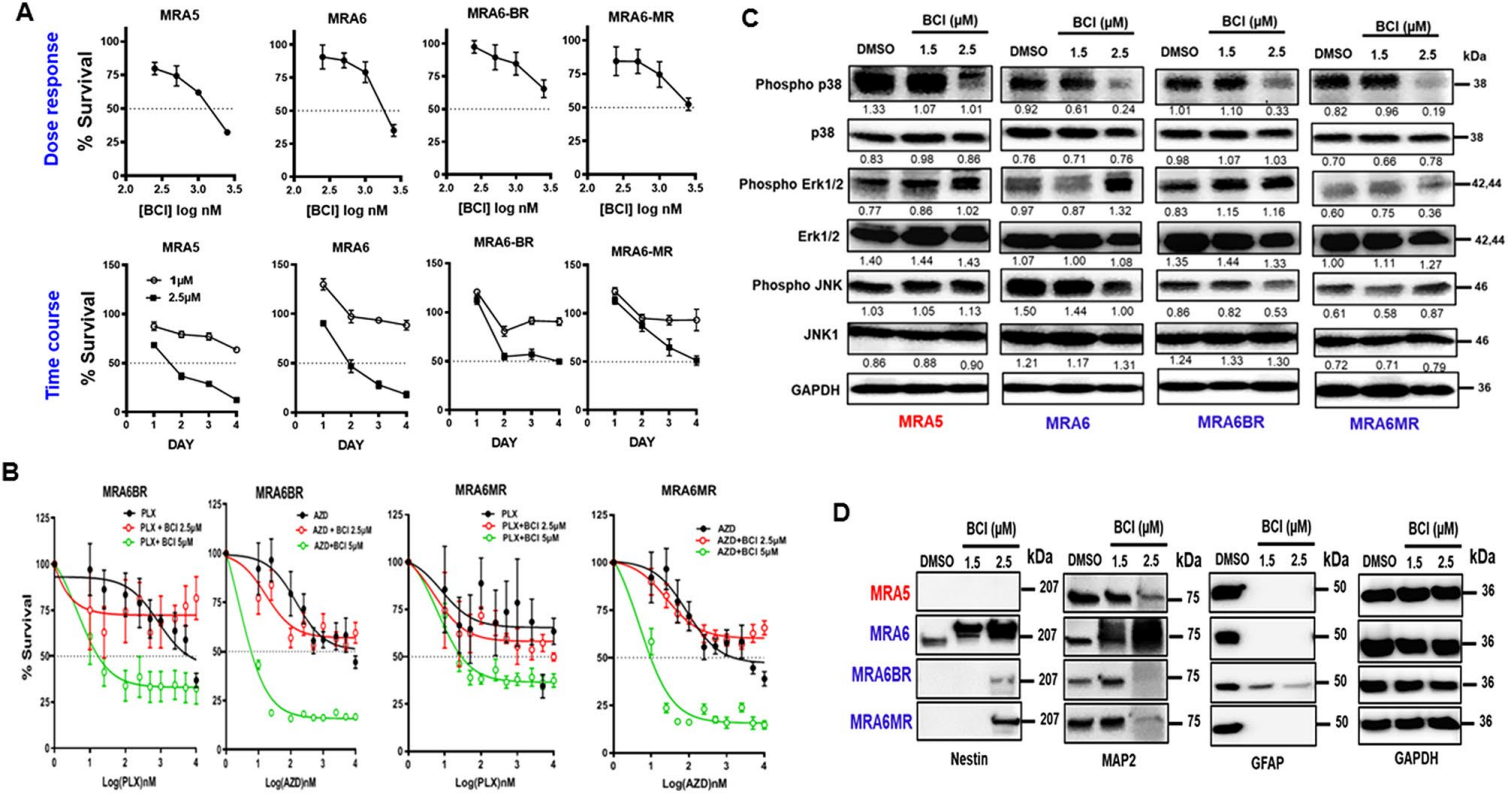
Effect of DUSP inhibitor on melanoma survival, differentiation and drug resistance. (**A**): (*Top panel*) Dose response curves for DUSP1/6 inhibitor BCI Cells were plated in 6 replicates in 96-well plates and treated with DMSO or increasing concentrations of BCI for 72 h. Cell survival was assessed using MTT assay. (*Bottom panel*) Time course of the effect of DUSP1/6 inhibition on the survival of melanoma cells. Cells were plated in 6 replicates in 96-well plates and treated with 1 µM and 2.5 µM BCI for 4 days. Cell survival was estimated using MTT assay. (**B**) Treatment with BCI sensitizes MAPKi-resistant MRA6BR and MRA6MR cells to BRAFi and MEKi, respectively. Cells in 6 replicate wells in 96-well plates were treated with increasing concentrations of BRAFi PLX4032 (PLX) or MEKi AZD6244 (AZD) alone or in combination with 2.5 µM or 5 µM BCI for 72 h and cell survival was assessed by MTT assay. The dotted line indicates 50% survival. (**C)** Western blot analysis of MAPK signaling protein in MAPKi-sensitive and MAPKi-resistant melanoma cells after a 24 h treatment with DMSO or 1.5 µM and 2.5 µM BCI. Numbers on the right indicate the molecular (kDa) of the respective proteins. Band intensities normalized to GAPDH expression using ImageJ are shown. (**D**) Western blot analysis of neural markers after a 24 h treatment with DMSO or 1.5 µM and 2.5 µM BCI. Numbers on the right indicate the molecular (kDa) of the respective proteins.

Next, we tested the effect of pharmacological inhibition of DUSP1/6 with BCI [(E)-2-benzylidene-3-(cyclohexylamino)-2,3-dihydro-1H-inden-1-one], a specific allosteric inhibitor of DUSP1/6 with EC_50_ of 13.3 μM and 8.0 μM for DUSP6 and DUSP1 in cells, respectively^31^. Treatment with BCI for 72 h reduced the survival of both MAPKi-resistant (MRA5) and sensitive (MRA-6) melanoma cell lines in a dose-dependent manner with an IC_50_ = 1–3 µM. However, MRA6BR, MRA6MR melanoma cells with acquired resistance were relatively less sensitive to BCI with an IC_50_ > 4 µM (Fig. 4A). Time course studies with 1 µM and 2.5 µM BCI showed that the survival of intrinsically MAPKi-resistant MRA5 cells was decreased as early as 24 h after treatment with both concentrations of BCI. However, the growth of MAPKi-sensitive MRA6 cells and its derivatives MRA6BR and MRA6MR with acquired resistance appeared to be initially stimulated after 24 h treatment (at 1 µM BCI) followed by a decrease in their survival upon prolonged treatment. Thus, intrinsically MAPKi-resistant MRA5 cells appear to be more sensitive DUSP1 inhibition than melanoma cells with acquired resistance.

We then asked whether BCI sensitizes BRAFi- and MEKi-resistant cells to killing by BRAFi and MEKi. We treated MRA6BR and MRA6MR cells with 10 nM–10 µM BRAFi or MEKi alone or together with 2.5 µM or 5 µM BCI for 3 days and assessed cell survival (Fig. 4B). Presence of 5 µM BCI markedly sensitized both BRAFi- and MEKi-resistant cells to BRAFi and MEKi (MEKi > BRAFi). These data show that DUSP1/6 inhibition can overcome MAPKi-resistance.

### DUSP 1/6 and phosphorylation of MAPKs

Next, we tested the effect of inhibition of DUSP1/6 by BCI on phosphorylation of MAP kinases. As shown in Fig. 4C, treatment with either 1.5 μM or 2.5 μM BCI treatment caused modest increase in Erk1/2 phosphorylation in all melanoma cells- MAPKi-sensitive MRA6, intrinsically MAPKi-resistant MRA5 and MRA6BR and MRA6MR cells with acquired MAPKi resistance. Interestingly, in the MRA6MR cell line, the level of Erk1/2 phosphorylation decreased with increasing BCI dose. Treatment with 2.5 μM BCI caused reproducible increase in JNK phosphorylation in MAPKi-resistant MRA5 and MRA6MR cells but decreased JNK phosphorylation in MAPKi-sensitive MRA6 cells and BRAFi-resistant MRA6BR cells. Similarly, treatment with either 1.5 or 2.5 µM BCI reduced p38 MAPK phosphorylation in all melanoma cell lines. These data suggest that DUSP1 regulates phosphorylation of ERK1/2 and p38 in melanoma cells.

Next, we asked if BCI treatment alters the expression of markers of neural differentiation. Treatment with increasing doses of BCI appeared to increase nestin expression in MAPKi-sensitive MRA6 cells, whereas BCI treatment induced nestin expression in MAPKi-resistant MRA6BR and MRA6MR cells (Fig. 4D). However, treatment with BCI did not induce nestin in intrinsically MAPKi-resistant MRA5 cells.

Treatment with 2.5 µM BCI resulted in downregulation of MAP2, a marker of immature precursor neurons, in MAPKi-resistant cells whereas there was a dose-dependent upregulation of MAP2 expression in MAPKi-sensitive MRA6 cells. In contrast, GFAP, which is expressed uniformly in all four cell lines, was downregulated by BCI in all cell lines except BRAFi-resistant MRA6 cells. These findings suggest a role for DUSP1 in plasticity of melanoma cells to neural differentiation and drug resistance.

### Significance of DUSPs in melanoma

To test the relationship of DUSPs to melanoma drug resistance, we queried Gene Expression Omnibus dataset. Analysis of the dataset GSE24862 consisting of mRNA expression in parental and PLX4032-resistant sub-lines derived from BRAF(V600E)-positive melanoma cell lines and short-term cultures from clinical trial patients^25^ showed that expression of mRNAs for DUSP4, -5, -6, -7 and -9 was higher (statistically significant with *p* values = 0.001–0.0292) in pre-treated samples compared to post-BRAFi treated samples showing that expression of several DUSPs is downregulated by BRAFi treatment (Supp Fig. S3A). Analysis of single cell RNA sequencing dataset GSE116237 consisting of pre- and post-BRAFi treated (days 4 and 28) patient tumor samples^26^ (Supp Fig. S3B) also showed that expression of DUSP4 and DUSP6 was progressively downregulated upon BRAFi treatment. Interestingly, analysis of neuronal differentiation markers showed that while there was a progressive increase in nestin, the neural stem cell marker, , there appeared to be a concomitant decrease in MAP2, a marker of neuronal precursor (Supp Fig. S3C).

**Figure 5:**
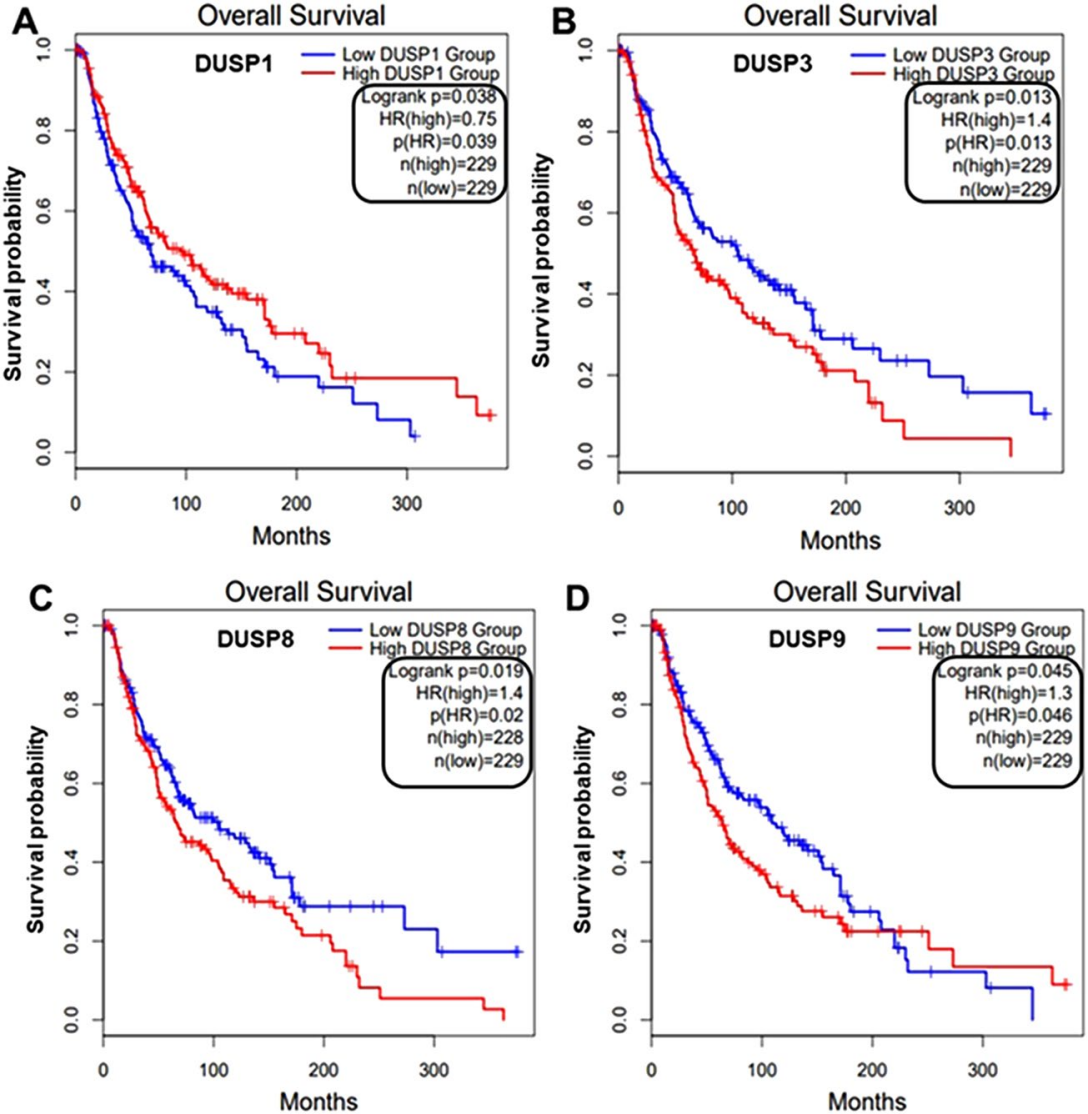
Prognostic significance of DUSPs in melanoma. Kaplan–Meier survival analysis of a cohort of melanoma patients in the TCGA-SKCM datasets for overall survival Patients were stratified based on the median expression (low- blue and high- red) of DUSP1, DUSP3, DUSP8, and DUSP9. Log rank test, hazard ratio (HR) are shown.

Next, to understand the prognostic significance of DUSPs in melanoma, we queried the TCGA-SKCM (Skin Cutaneous Melanoma) datasets for DUSP mRNA expression using GEPIA2, an online tool for determining overall survival (OS) (Fig. 5 and Supp Fig. 4). This dataset consists of melanoma tumors that include (a) both primary and metastatic tumors, (b) tumors that harbor known oncogenic drivers and those with no identified oncogenic driver, and (c) with or without treatment. In this diverse set of samples, we found that high DUSP1 (HR = 0.0.75, *p* = 0.039) mRNA expression was associated with better survival whereas high mRNA of DUSP3 (HR = 1.4, *p* = 0.013), DUSP8 (HR = 1.4, *p* = 0.02) and DUSP9 (HR = 1.3, *p* = 0.046) was associated with poor survival.

Additionally, a query of a database generated recently by a systematic study of druggable genes in cancer revealed that DUSP1 has the highest potential as a drug target among all DUSPs (Table S3).

In summary, our study shows a relationship between DUSPs, MAPKi-resistance and neuronal differentiation of melanoma, and suggest that targeting DUSPs is a potential strategy to overcome MAPKi-resistance in melanoma.

## Discussion

Cellular plasticity and disrupted differentiation including transdifferentiation are now considered hallmarks of cancer^3^. It now recognized that phenotypic plasticity of cancer cells and their differentiation along diverse cellular states also contributes to the acquisition of resistance to targeted therapy in various cancers^2^. However, genomic and non-genomic changes associated with drug resistance could also modify cellular plasticity and cell fates^32^. Melanoma is known to differentiate along multiple cellular pathways, including endothelial (also known as vasculogenic mimicry) and neuronal cell types^3,8,33^. Melanoma differentiation and transdifferentiation has also been implicated as a mechanism for drug resistance^34^.

In this study, we found that MAPKi resistance is associated with a decrease in the expression of the neural crest stem cell marker nestin as well as a decrease in the expression of the neuronal precursor marker MAP2. In melanoma cells with acquired MAPKi-resistance, BRAFi and MEKi downregulate DUSP1, and knockdown or inhibition of DUSP1 alone can overcome both intrinsic and acquired MAPKi-resistance. Query of gene expression data from pre- and post-BRAFi treated patient derived xenograft studies showed that among all expressed DUSPs. DUSP1 mRNA exhibits the most variable expression between pre- and post-BRAFi treated resistant tumors. Query of TCGA melanoma dataset showed that high DUSP1 mRNA expression is associated with better overall survival.

Resistance, both intrinsic and acquired, to MAPK pathway targeted treatments, continues to pose a challenge in management of melanoma. Intrinsic resistance, i. e., lack of response to therapy occurs less frequently (in 10% of BRAF-mutated melanomas) and is thought to be associated with PTEN and MAP2K1^35^. Despite the quick response rates observed with combination of BRAFi and MEKi in patients with BRAF mutant tumors, resistance develops frequently^36^. Several mechanisms have been identified that contribute to acquired resistance. These include amplification of the mutant BRAF gene*,* upregulation of receptor tyrosine kinases or NRAS or Src kinase signaling^25,37,38^. Patients who do not respond to BRAFi monotherapy also do not respond to MEKi indicating cross-resistance and tumor heterogeneity in the context of mechanisms of drug resistance^39,40^. Indeed, genomic investigations of pre- and post-treatment samples from patients treated with BRAFi suggest that distinct resistance mechanisms within and between tumors in the same patient^41^. Therefore, both genomic (gene amplification and deletion) and non-genomic (including gene expression changes and epigenetic modifications) mechanisms are known to contribute MAPKi resistance in melanoma^1,42–45^.

We previously demonstrated that MAP2, a neuron-specific protein, is abundantly expressed in early invasive primary melanoma lesions and primary melanoma cell lines but is absent in metastatic melanoma lesions and cell lines^10^. MAP2 exists in three developmentally regulated alternatively spliced isoforms. These are the mature MAP2a and b (280 kDa) and the juvenile MAP2c (75 kDa). Whereas the juvenile MAP2c form is only found in immature neurons, the mature MAP2ab forms are found in neurons throughout their lives^46^. Interestingly, we found that the mature MAP2, a marker for neuronal terminal differentiation, is abundantly expressed in primary melanoma showing that melanoma cells can fully transdifferentiate along neuronal linage^10^.

In recent studies, we found that melanoma neural differentiation is associated with the development of MAPKi resistance without prior drug treatment reminiscent of intrinsic resistance^13^. Interestingly, the plasticity of metastatic melanoma cells was enhanced by BRAFi and melanoma cells that acquire plasticity have a propensity to transdifferentiate to neural lineages and exhibit MAPKi resistance^13^. In the current study, we discovered that expression of the juvenile MAP2c form was present in both MAPKi-resistant and -sensitive cells, regardless of BRAFi/MEKi treatment, whereas expression of nestin, a marker of NCSC, is only found in MAPKi-sensitive cells and not in MAPKi-resistant cells. These findings support our hypothesis that neuronal differentiation in melanoma is linked to the development of MAPKi resistance, with or without prior drug treatment.

While treatment with MAPK inhibitors effectively inhibits MAPK signaling, in some cases it may alter negative feedback loops, resulting in pathway reactivation. The induction of negative regulators such as Sprouty (Spry) and dual specificity phosphatases (DUSPs) by extracellular signal-regulated kinase (ERK) can prevent excessive stimulation of Ras/mitogen-activated protein kinase (MAPK) signaling in physiological conditions. This is also evident in our experiments where although treatment of MAPKi-resistant (MRA6BR and MRAMR cells acquired resistance) cells with BRAFi (but not combination of BRAFi and MEKi) caused downregulation of DUSP1, this downregulation in the presence of BRAFi was not sufficient to decrease the survival of these MAPKi-resistant cells (Fig. 1C). On the other hand, DUSP1 KD alone (in the absence of BRAFi) decreased the survival of MAPK-resistant cells.

Mutant BRAF activates the pathway downstream of Ras abnormally in melanomas, bypassing this feedback loop. Inhibitors such as vemurafenib prevent ERK activation, resulting in Spry and DUSP downregulation^47^. In this study, we found that DUSP-1, -3, -8, and -9 expression is downregulated in advanced metastatic, but not early lymph node metastatic, melanoma cells. Amon the advanced metastatic cell lines, DUSPs appear to be regulated by MAPK pathway only in MAPKi-sensitive and -(acquired) resistant cells but not in intrinsically MAPKi resistant cells. These data suggest that regulation of DUSP by MAPK signaling is a feature of advanced metastatic melanoma.

Interestingly, DUSPs are negative regulators of MAPK signaling and are known to regulate pluripotency and stemness of cancers^20,21^. In the context of neural differentiation, DUSP1 is of interest because studies on *Dusp1*^*-/-*^ mice showed that Dusp1 is required to maintain neuronal survival and differentiation^22,23^. DUSP1 is an inducible nuclear phosphatase with a target specificity that varies depending on the cellular context^17,24^. In this study, we found that treatment of melanoma cells with BCI, a DUSP1/DUSP6 inhibitor at a concentration close to IC50 for selective inhibition of DUSP1, caused upregulation of nestin and immature MAP2 in MAPKi-sensitive cells. DUSP1 inhibition had no effect on the glial marker GFAP suggesting that DUSP1 (and/or DUSP6) is involved in melanoma transdifferentiation and emergence of neural lineages.

Our analysis of available melanoma gene expression studies of melanoma tumors and patient-derived xenografts pre- and post-BRAFi (resistant) showed that several DUSPs, including DUSP1, are downregulated in the resistant tumors (both in bulk and single cell RNAseq datasets). In a recent genome-wide druggable target analysis, among all DUSPs, DUSP1 emerged as a druggable target with high potential. In summary our data implicate DUSPs, specifically DUSP1, as mechanistic link between melanoma plasticity, tendency to differentiate along neural linages and emergence of MAPKi resistance phenotype. Additional studies are warranted on the role of DUSPs, in general and DUSP1 in particular, in melanoma drug resistance and their potential as targets for treatment of melanoma.

## Supplementary Information

Supplementary Information.
