# Supplementary material for: Role of dual specificity phosphatases (DUSPs) in melanoma cellular plasticity and drug resistance
Source: Sci Rep. 2022 Aug 23;12:14395. doi: 10.1038/s41598-022-18578-x (PMC9399232; doi:10.1038/s41598-022-18578-x)

Supplementary Material

Role of Dual Specificity Phosphatases DUSPs in Melanoma Cellular Plasticity and Drug Resistance

Mithalesh K. Singh[1, §], Sarah Altameemi[1], Marcos Lares[1], Michael A. Newton[3] and Vijayasaradhi Setaluri [1,2,§]

Department of Dermatology, School of Medicine and Public Health, University of Wisconsin-Madison, William S. Middleton Memorial Veterans Hospital, Madison, WI USA

[1]Department of Dermatology, University of Wisconsin School of Medicine and Public Health,

University of Wisconsin-Madison, WI, 53705

[2] William S. Middleton Memorial Veterans Hospital, Madison, WI, 53705

[3] Department of Statistics, Department of Biostatistics and Medical Informatics, University of Wisconsin-Madison, Madison, WI 53706, USA

§ Address for Correspondence: Vijayasaradhi Setaluri, PhD and Mithalesh K. Singh, PhD

Department of Dermatology, Wisconsin Institute for Medical Research, 1111 Highland Avenue, University of Wisconsin-Madison, Madison, WI, 53706; Phone: (608) 263-5362

e-mail: vsetaluri@dermatology.wisc.edu; mksingh6@wisc.edu; mithales@hs.uci.edu

Conflict of Interest: The authors do not have any conflict of interest and do not have any disclosures

Supplementary Tables:

Table S1: Melanoma cell lines used in this study

Cell Line	Stage	Stage/Level	Mutations				Homozygous Loss
			BRAF	PTEN	N-Ras	c-KIT	
WM115	VGP	Level III	V600D	Hemizygous Deletion	WT	WT	CDKN2A, CDKN2B
WM266-4	Metastasis	Level III	V600D	Hemizygous Deletion	WT	WT	CDKN2A, CDKN2B
WM239A	Metastasis	Level III	V600D	Hemizygous Deletion	WT	WT	CDKN2A, CDKN2B
WM165-1	Metastasis	Level III	V600D	Hemizygous Deletion	WT	WT	CDKN2B
MRA-5	Metastasis	Stage IV	V600E	Absent	ND	ND	ND
MRA-6	Metastasis	Stage IV	V600E	Absent	ND	ND	ND

Data for the cell lines highlighted in light blue were obtained from Rockland Immunochemicals Inc., Limerick, PA. MRA series of cell lines were established in-house by Dr. Mark Albertini and the gene mutation data were obtained targeted sequencing and western blotting.
RGP: radial growth phase; VGP: vertical growth phase; Stage: AJCC stage; Level: Clark's level;
WT: wild type;
Absent: protein not detected by immunoblotting assay

Table S2: Sources and dilutions of antibodies used in this study

Antibodies	Catalogue #	Dilution	Source	Vendor
DUSP1/MKP1	ab236501	1:750	Mouse	
DUSP3	ab125077	1:750	Rabbit	
DUSP8	ab198175	1:750	Rabbit	
DUSP9	ab194355	1:750	Rabbit	Abcam
Nestin	33475S	1:1000	Mouse	
MAP2	8707S	1:1000	Rabbit	
GFAP	3670S	1:1000	Mouse	
phospho-p38 MAPK (Thr180/Tyr182)	4511S	1:1000	Rabbit	
p38 MAPK	8690S	1:1000	Rabbit	
p44/42 Map Kinase (Erk1/2)	4696S	1:1000	Mouse	
Phospho-p44/42 MAPK (Erk1/2)	4370	1:1000	Rabbit	
phospho-SAPK/JNK (Thr183/Tyr185)	9255S	1:750	Mouse	
JNK1	3708S	1:1000	Mouse	Cell Signaling Technology
GAPDH	6000-1-Ig	1:1500	Mouse	Proteintech
Anti-Rabbit HRP-conjugated	NA934	1:2000	Donkey	
Anti-Mouse HRP-conjugated	NA931	1:2000	Goat	GE Healthcare

Table S3: List of all DUSPs genes with the potential druggable targets using TCDA (The Cancer Druggable Gene Atlas)

Genes	Functional class	PubTator score	Target development /druggability level (TDL)	Tractability (smallmolecule)	Tractability (antibody)
BRAF	Kinase	99%	Tclin	Clinical precedence	Predicted Tractable-High confidence
DUSP1	Enzyme	90%	Tchem	Discovery precedence	-
DUSP2	Enzyme	86%	Tbio	Predicted Tractable	-
DUSP3	Enzyme	79%	Tchem	Discovery precedence	
DUSP4	Enzyme	71%	Tbio	NA	-
DUSP5	Enzyme	62%	Tbio	Predicted Tractable	-
DUSP6	Enzyme	80%	Tbio	Discovery precedence	-
DUSP7	Enzyme	43%	Tbio	NA	
DUSP8	Enzyme	48%	Tbio	NA	-
DUSP9	Enzyme	47%	Tbio	NA	
DUSP10	Enzyme	56%	Tbio	NA	
DUSP11	Enzyme	47%	Tbio	NA	
DUSP12	Enzyme	49%	Tbio	NA	-
DUSP14	Enzyme	33%	Tbio	NA	-
DUSP15	Enzyme	33%	Tbio	Discovery precedence	Predicted Tractable
DUSP16	Enzyme	51%	Tbio	NA	-
DUSP18	Enzyme	34%	Tbio	NA	-
DUSP19	Enzyme	27%	Tdark	NA	-
DUSP20	Enzyme	-	-	-	-
DUSP22	Enzyme	30%	Tbio	Discovery precedence	Predicted Tractable
DUSP23	Enzyme	51%	Tbio	Discovery precedence	-
DUSP28	Enzyme	25%	Tbio	NA	

Tclin:- Potential druggable genes were targeted by approved drugs
Tchem:- small molecules that satisfy the activity thresholds
Tbio/Tdark: biological functions were still unknown
http://fcgportal.org/TCDA/gene_detail.php

SUPPLEMENTARY FIGURE LEGENDS

Supple Figure S1: Gene Expression analysis of DUSPs in MAPKi-resistant (MRA5, MRA6BR, and MRA6MR) and MAPKi-sensitive (MRA6) BRAFV600E mutant cells and genetically matched cell lines established from same patients. (A) RNA sequencing for DUSP genes in MAPKi-resistant (MRA5, MRA6BR, and MRA6MR) and MAPKi-sensitive (MRA6) BRAFV600E mutant cells. For six DUSP genes, z-scores were computed. The differential expression of all six DUSPs is represented in the form of a heatmap, demonstrating their differential effect of expression from MAPKi-sensitive to MAPKi-resistant cells. **(A)** RNA sequencing differential expression data for DUSP genes in genetically matched primary (**P**) WM115 and lymph node metastatic cell lines WM239A (**L1**), WM266-4 (**L2**) and WM165-1 (**L3**)**.** For all twenty DUSP genes, z-scores were computed. The differential expression of all six DUSPs is represented in the form of a heatmap, demonstrating their differential effect of expression from **P** to **L3** cells.

Supple Figure 2: Effect of DUSP1 KD and DUSP8 KD on sensitivity of melanoma cells to MAPKi. (A) The percentage of DUSP1 and DUSP8 knockdown was calculated using ImageJ analysis and normalized with GAPDH in MAPKi-resistant MRA5, MRA6BR, MRA6MR, and MAPKi-sensitive MRA6 cells. **(B)** MAPKi-resistant MRA5, MRA6BR, MRA6MR and MAPKi-sensitive MRA6 cells were transfected with DUSP1 and DUSP8 siRNAs. Cells were trypsinized 24h post-transfection and 5000 cells were seeded in 5-6 replicate wells in 96-well plates and treated for 48h with DMSO or 2.5µM PLX4032, AZD6244 or 10µM PLX4032, AZD6244 (MRA5 and MRA6 cells). or combination of PLX-4032+AZD-6244 (MRA5, MRA6, MRA6BR and MRA6MR cells). All datas are shown as mean ± SD and analyzed using Student's t-test. P values: * denotes $P \leq 0.05$, ** ≤ 0.01; *** ≤ 0.001 and **** ≤ 0.0001.

Supple Figure 3: Gene expression analysis of DUSPs in publicly available datasets. (A) GEO dataset GSE24862 consists of parental and resistant sub-lines of melanoma cell lines treated or untreated with PLX4032 Log base 2 transformed expression (Affymetrix Human Gene 1.0 ST Array) is shown for 18 DUSP genes and for 12 DMSO treated and 12 PLX treated samples. Genes are ordered from top to bottom by fold change (difference in average log expression between two treatment groups). The observed expression changes are statistically significant (Benjamini-Hochberg adjusted p-value < 0.01) for DUSP4,5,6,7, according to the Wilcoxon rank sum test. **(B)** GSE116237 datasets consists of single cell RNA and single cell DNA sequencing from a PDX melanoma model before and on treatment (BRAF&MEK inhibitors)**.** Log base 2 transformed expression (Affymetrix Human Gene 1.0 ST Array) is shown for 7 DUSP genes. Genes are ordered from left to right by fold change [difference in average log expression between untreated (T0) vs four-day treatment (T4) vs 28-day treatment (T28)]. The observed expression changes are statistically significant (Benjamini-Hochberg adjusted p-value < 0.01) according to the Wilcoxon rank sum test. **(C)** GSE116237 datasets consists of single cell RNA and single cell DNA sequencing from a PDX melanoma model before and on treatment (BRAF&MEK inhibitors)**.** Log base 2 transformed expression (Affymetrix Human Gene 1.0 ST Array) is shown for Nestin and MAP2 genes. Difference in average log expression between untreated (T0) vs four-day treatment (T4) vs 28-day treatment (T28)]. The observed expression changes are statistically significant (Benjamini-Hochberg adjusted p-value < 0.01) according to the Wilcoxon rank sum test.

Supple Figure 4: Prognostic significance of DUSPs in BRAF mutant melanoma. Kaplan-Meier survival analysis for overall survival of a cohort of melanoma patients with BRAF mutation in the TCGA-SKCM datasets. Patients were divided into two categories based on the

median expression of DUSP1, DUSP3, DUSP8, and DUSP9. The log rank test and hazard ratio (HR) are displayed.

Supplementary Figure 1

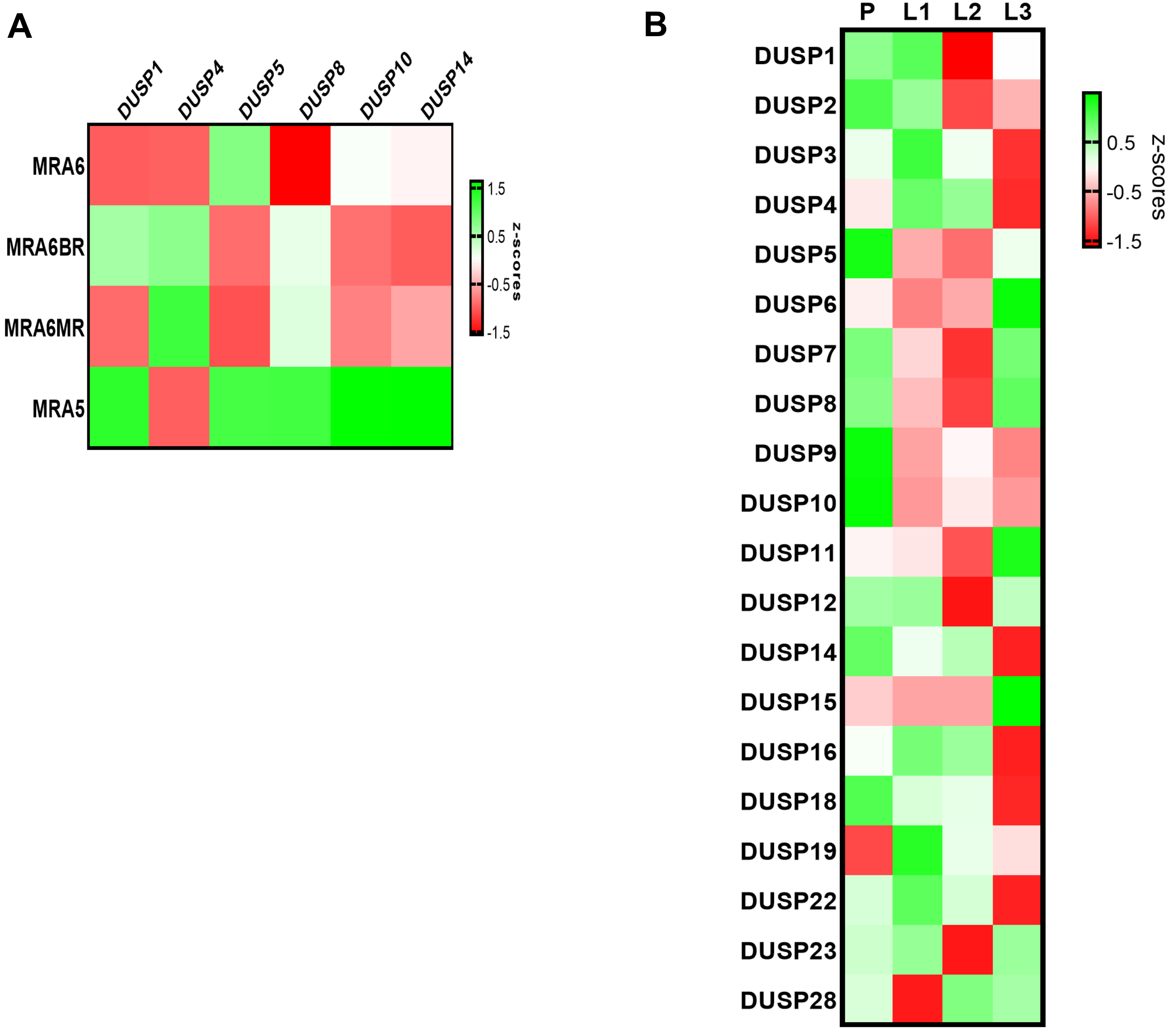

Supplementary Figure 2

A

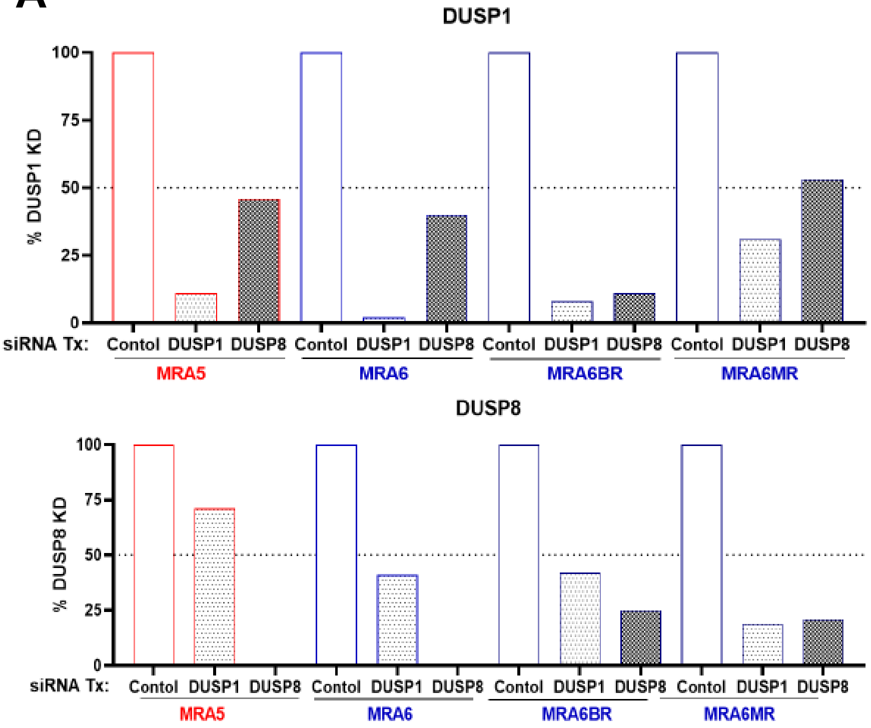

B

Cell Lines	siRNA Transfection	Comparison	p-value
MRA5	Control	DMSO vs BRAFi	****
		DMSO vs MEKi	****
	DUSP1	DMSO vs BRAFi	****
		DMSO vs MEKi	****
	DUSP8	DMSO vs BRAFi	****
		DMSO vs MEKi	****
MRA6	Control	DMSO vs BRAFi	****
		DMSO vs MEKi	****
	DUSP1	DMSO vs BRAFi	****
		DMSO vs MEKi	****
	DUSP8	DMSO vs BRAFi	****
		DMSO vs MEKi	****
MRA6BR	Control	DMSO vs BRAFi	****
		DMSO vs MEKi	****
	DUSP1	DMSO vs BRAFi	****
		DMSO vs MEKi	****
	DUSP8	DMSO vs BRAFi	****
		DMSO vs MEKi	****
MRA6MR	Control	DMSO vs BRAFi	****
		DMSO vs MEKi	****
	DUSP1	DMSO vs BRAFi	****
		DMSO vs MEKi	****
	DUSP8	DMSO vs BRAFi	****
		DMSO vs MEKi	****

C

Cell Lines	siRNA transfection	Comparison	p-value
MRA5	Control	DMSO vs BRAFi+MEKi	****
	DUSP1	DMSO vs BRAFi+MEKi	****
	DUSP8	DMSO vs BRAFi+MEKi	****
MRA6	Control	DMSO vs BRAFi+MEKi	****
	DUSP1	DMSO vs BRAFi+MEKi	****
	DUSP8	DMSO vs BRAFi+MEKi	****
MRA6BR	Control	DMSO vs BRAFi+MEKi	****
	DUSP1	DMSO vs BRAFi+MEKi	****
	DUSP8	DMSO vs BRAFi+MEKi	****
MRA6MR	Control	DMSO vs BRAFi+MEKi	****
	DUSP1	DMSO vs BRAFi+MEKi	****
	DUSP8	DMSO vs BRAFi+MEKi	****

Supplementary Figure 3

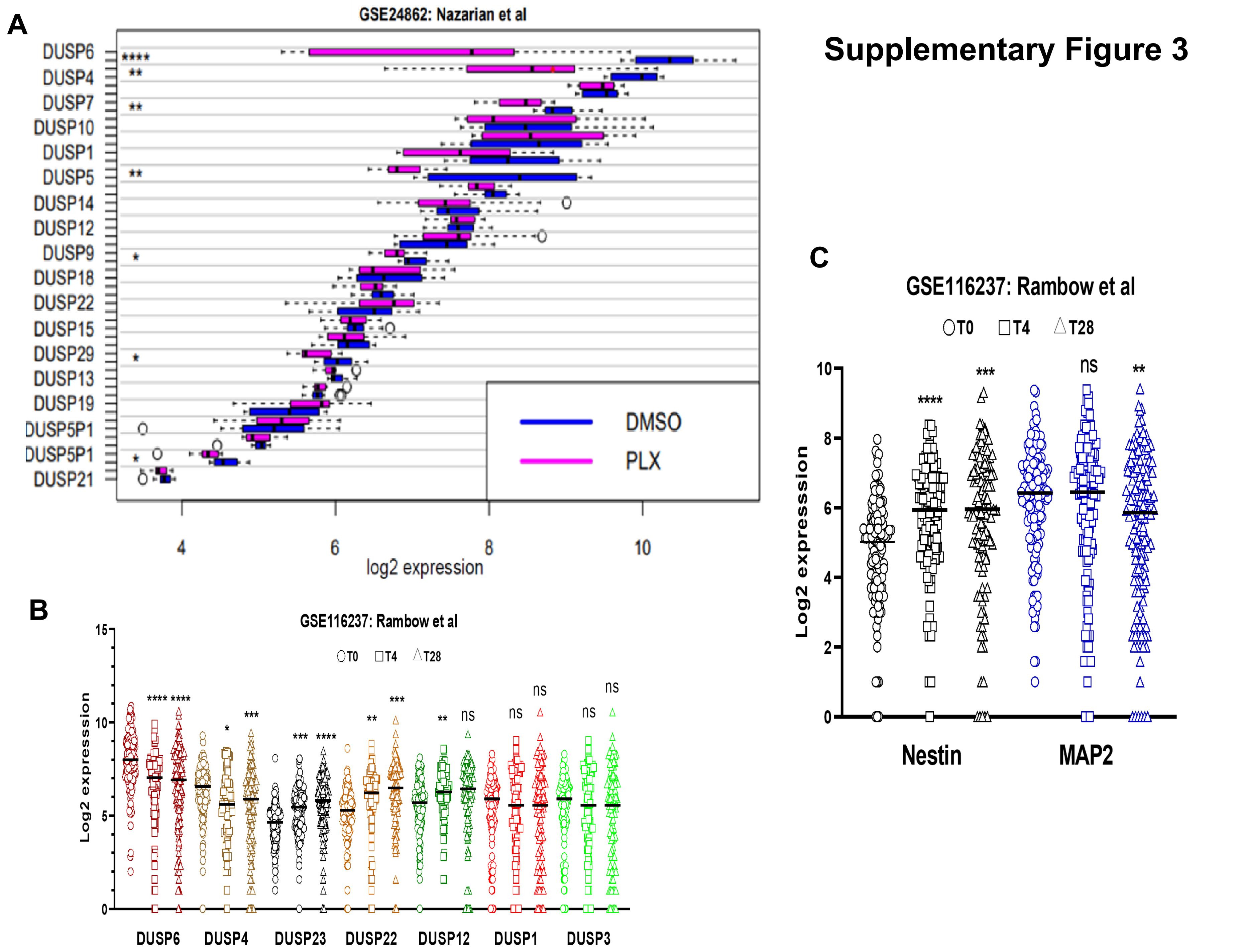

Supplementary Figure 4

Melanoma patients (n=144) with BRAF mutations from the TCGA-SKCM dataset (n=458)

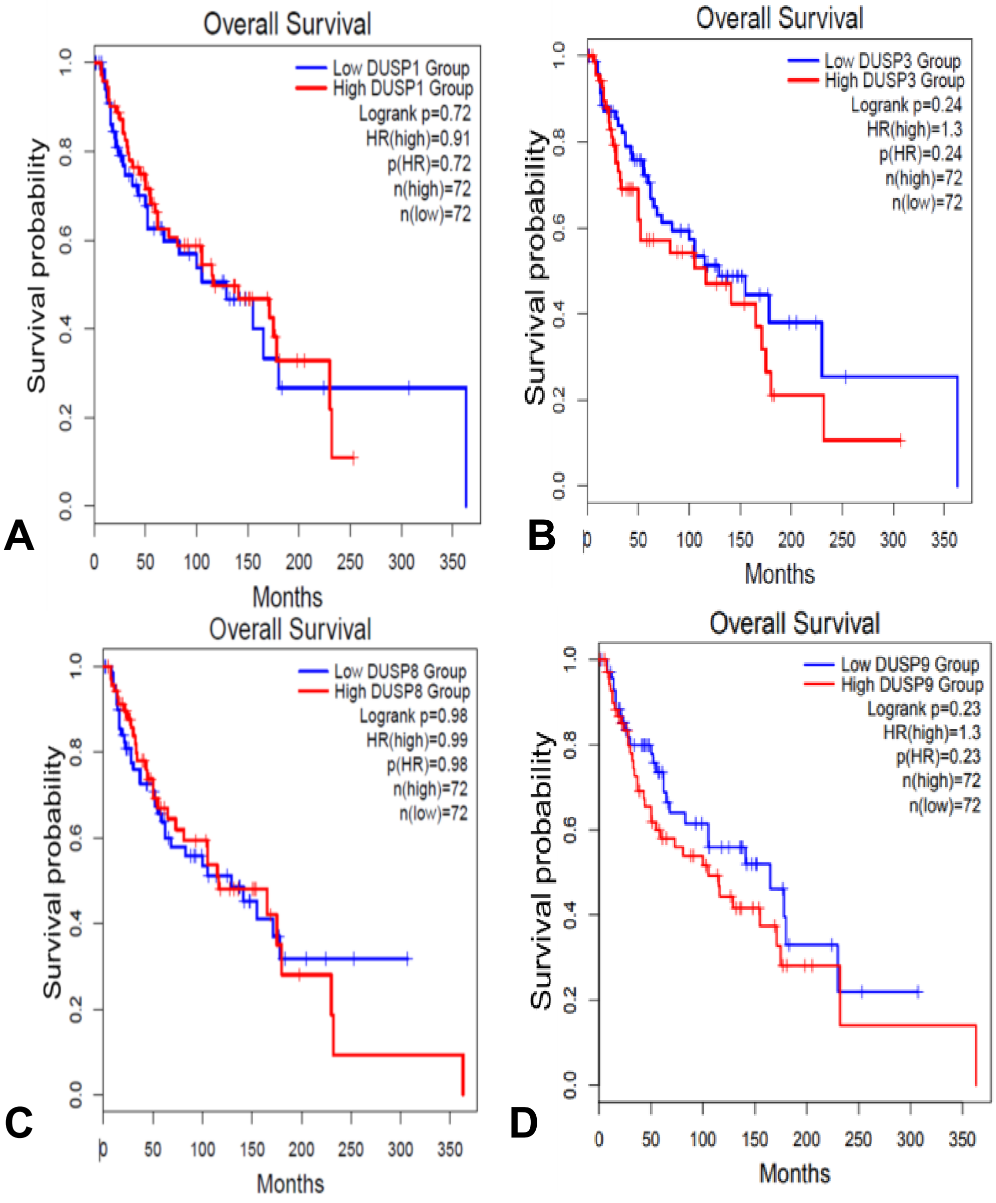

Supplement: Supplementary file 1 — Supplementary Information. [file 41598_2022_18578_MOESM1_ESM.pdf]
